# Fermentation supernatants of *Lactobacillus delbrueckii* inhibit growth of human colon cancer cells and induce apoptosis through a caspase 3-dependent pathway

**DOI:** 10.3892/ol.2014.1959

**Published:** 2014-03-10

**Authors:** YING WAN, YI XIN, CUILI ZHANG, DACHANG WU, DAPENG DING, LI TANG, LAWRENCE OWUSU, JING BAI, WEILING LI

**Affiliations:** 1 Department of Biotechnology, Dalian Medical University, Dalian, Liaoning 116044, P.R. China; 2 Clinical Laboratory Department, The First Affiliated Hospital of Dalian Medical University, Dalian, Liaoning 116044, P.R. China

**Keywords:** *Lactobacillus delbrueckii*, colon cancer, apoptosis, caspase 3, matrix metalloproteinase-9

## Abstract

Probiotic bacteria are known to exert a wide range of beneficial effects on their animal hosts. Therefore, the present study explored the effect of the supernatants obtained from *Lactobacillus delbrueckii* fermentation (LBF) on colon cancer. The results indicated that the proliferation of LBF solution-treated colon cancer SW620 cells was arrested and accumulated in the G1 phase in a concentration-dependent manner. The LBF solution efficiently induced apoptosis through the intrinsic caspase 3-depedent pathway, with a corresponding decreased expression of Bcl-2. The activity of matrix metalloproteinase 9, which is associated with the invasion of colon cancer cells, was also decreased in the LBF-treated cells. In conclusion, the results demonstrate the antitumor effect of LBF *in vitro* and may contribute to the development of novel therapies for the treatment of colon cancer.

## Introduction

Colon cancer is the third and fourth most common type of cancer in females and males worldwide, respectively, with >550,000 new cases and ~300,000 mortalities reported in 2008. Over the past 25 years, approximately one million individuals worldwide have been diagnosed with colon cancer annually (1). The risk factors of colorectal cancer are varied and include changes in the bacterial community of the colon (2).

A total of ~100 trillion bacteria colonize the human gastrointestinal tract and the colon is estimated to be populated with 10^14^ types of bacteria (3). It is generally accepted that these bacteria are not harmful, but extremely beneficial for the individual (4), and the modulation of the bacterial community in the colon presents an interesting approach to improve health. Furthermore, Rafter (5) reported that probiotic intake may modulate the bacterial community of the colon and reduce colon cancer incidence.

Lactobacillus is a type of probiotic which colonizes the human gastrointestinal tract, and reports have indicated that certain Lactobacillus strains exhibit tumor-suppressing properties (6). *Lactobacillus delbrueckii* is a strain of Lactobacillus used for the production of yogurt, a study has shown that the yogurt fermented by *L. delbrueckii* reduces the formation of colonic aberrant crypt foci in transgenic rats (7). However, whether *L. delbrueckii* inhibits the growth of colon cancer and the underlying mechanisms of this process remain unknown.

Therefore, the aim of the present study was to evaluate whether the supernatants obtained from *L. delbrueckii* fermentation (LBF) have the capacity to inhibit the proliferation and growth of colon cancer cells, as well as to investigate the underlying mechanisms of this.

## Materials and methods

### Preparation of LBF solution

*L. delbrueckii* was obtained from the American Type Culture Collection [ATCC (Manassas, VA, USA) and fermented in de Man, Rogosa and Sharpe medium (Sigma-Aldrich, St. Louis, MO, USA) at 37°C for 24 h. The supernatant fluid was acquired by centrifugation (3,469 × g for 5 min) and stored at −20°C as LBF stock solution.

### Cell culture of SW620 cells

The human colon cancer SW620 cell line was obtained from the ATCC and cultured using L-15 medium (Thermo Labsystems, Milford, MA, USA) containing 2 mmol/l L-glutamine and 2 g/l sodium bicarbonate, supplemented with antibiotics (100 U/ml penicillin and 100 mg/ml streptomycin) and 10% fetal bovine serum (FBS) purchased from Gibco-BRL (Carlsbad, CA, USA)]. The cells were maintained at 37°C in a humidified atmosphere of 5% CO_2_.

### Cell viability assay

The growth inhibitory effect of the LBF solution on SW620 cells was examined using 3-(4,5-dimethylthiazol-2-yl)-2, 5-diphenyltetrazolium bromide (MTT; Sigma-Aldrich) assay. The SW620 cells (5×10^5^ cells/ml) were seeded into 96-well plates and incubated for 24 h. Next, 20 μl LBF solution containing various concentrations of total protein (0, 0.025, 0.038, 0.05, 0.063, 0.076, 0.1, 0.2, 0.25, 0.4, 0.6, 0.625 and 0.75 mg/ml) was added to each well. The negative control group was treated with phosphate-buffered saline (PBS; Thermo Labsystems) buffer. Each concentration of LBF solution was repeated in five wells. Following 24 h of LBF solution treatment, 20 μl MTT solution (5 mg/ml) was added into each well and incubated for an additional 4 h. Subsequently, 100 μl dimethyl sulfoxide was added to each well and the absorbance values of the wells were measured at a wavelength of 492 nm using a Multiskan Ascent plate reader (Thermo Labsystems).

### Cell cycle analysis and Annexin V/propidium iodide (PI) staining assay

The SW620 cells (3×10^6^ cells/ml) were seeded into six-well plates and treated with 0.25 mg/ml LBF solution for 24 h. Following treatment, the cells were harvested and washed twice with PBS. For the cell cycle analysis, the cells were fixed in 70% ethanol overnight at 4°C. The fixed cells were then stained with PI solution (Sigma-Aldrich), which contained RNase A, for 45 min in the dark and analyzed by flow cytometry. For the Annexin V/PI staining assay, the cells were stained with Annexin V and PI solution for 10 min in the dark and analyzed by flow cytometry (Becton-Dickinson and Company, Franklin Lakes, NJ, USA). The untreated cells were used as a negative control.

### Immunohistochemistry

The SW620 cells (6×10^4^ cells/ml) were seeded into six-well plates and treated with 0.25 mg/ml LBF solution for 24 h. The cell monolayer was fixed and treated with 0.5% Triton X-100 (Sigma-Aldrich) for 20 min and 3% H_2_O_2_ for 15 min. Following blocking with 10% FBS/PBS, primary mouse anti-human caspase 3 polyclonal antibody and rabbit anti-human Bcl-2 polyclonal antibody (1:100; Santa Cruz Biotechnology, Inc., Santa Cruz, CA, USA) were added and incubated overnight at 4°C, followed by incubation with the goat anti-mouse and goat anti-rabbit, polyclonal, secondary antibody (Santa Cruz Biotechnology, Inc.) at a dilution of 1:200 for 30 min. The sections were visualized by 3-3′-diaminobenzidine (Roche Diagnostics GmbH, Mannheim, Germany) and the untreated cells were used as a negative control.

### Western blot analysis

The SW620 cells treated with 0.25 mg/ml LBF solution for 24 h were collected by centrifugation at 2,220 × g for 5 min at 4°C. The cells were then lysed in radioimmunoprecipitation assay buffer (Santa Cruz Biotechnology, Inc.) and 50 μg of total protein was separated on 10% sodium dodecyl sulfate polyacrylamide gel electrophoresis gels (Sigma-Aldrich) for 2 h. Next, the separated proteins were transferred onto nitrocellulose membranes (Pall Corporation, Port Washington, NY, USA) by semi-dry apparatus (Bio-Rad, Hercules, CA, USA) for 1 h, followed by blocking with 5% non-fat milk for 1 h. Subsequently, the specific primary mouse anti-human caspase 3 polyclonal (1:1,000), rabbit anti-human Bcl-2 polyclonal (1:1,000) and mouse anti-human β-actin polyclonal (1:10,000) antibodies were added at optimized dilutions (Santa Cruz Biotechnology, Inc.) and incubated overnight at 4°C. Following incubation with the secondary conjugated with HRP goat anti-mouse and goat anti-rabbit antibodies (1:10,000) for 1 h, the protein bands were visualized by an enhanced chemiluminescence kit (western Blotting Luminol reagent; Santa Cruz Biotechnology, Inc.).

### Gelatin zymography assay

The SW620 cells treated with 0.25 mg/ml LBF solution for 24 h and the culture medium were collected by centrifugation at 555 × g for 5 min at 4°C. Next, 20 μl of cell culture medium was electrophoresed on non-denaturing 10% polyacrylamide gels containing 0.1% gelatin (Sigma-Aldrich). Following electrophoresis, the gels were soaked in 2.5% Triton X-100 for 45 min and then incubated in substrate buffer (50 mM Tris-HCl, pH 7.5; 5 mM CaCl_2;_ and 0.02% NaN_3_) for 18 h. Subsequently, the gels were stained with 0.05% Coomassie brilliant blue G250 (Sigma-Aldrich) and destained in 10% acetic acid and 20% methanol.

### Statistical analysis

Data are presented as the mean ± standard deviation. Statistical analyses were performed using SPSS 11.5 software (SPSS, Inc., Chicago, IL, USA) and one-way analysis of variance with Bonferroni’s multiple comparison test was used to evaluate the differences between the different treatment groups. P<0.01 was considered to indicate a statistically significant difference.

## Results

### LBF solution inhibits the proliferation of colon cancer cells

**Figure 1:**
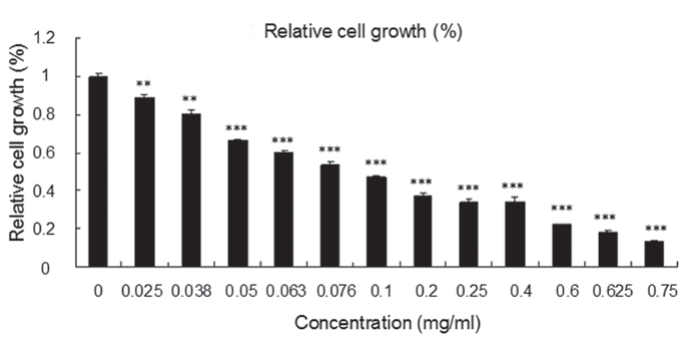
Effect of LBF solution on the proliferation of colon cancer SW620 cells. The SW620 cells were treated with various concentrations of LBF solution (0, 0.025, 0.038, 0.05, 0.063, 0.076, 0.1, 0.2, 0.25, 0.4, 0.6, 0.625 and 0.75 mg/ml) for 24 h. Growth is expressed as relative to untreated control cells. Data are presented as the mean ± SEM, from three independent experiments. One-way analysis of variance with Bonferroni’s multiple comparison test was used for statistical analysis.^**^P<0.01 and ^***^P<0.001, vs. untreated control cells. LBF*, Lactobacillus delbrueckii* fermentation.

The inhibitory effect of LBF solution on SW620 cells was detected by MTT assay for 24 h. Compared with the control group, treatment with LBF solution was found to significantly inhibit the cellular proliferation of SW620 cells with an IC_50_ value of 0.25 mg/ml after 24 h (P<0.001). The results showed that cells treated with LBF solution exhibit a markedly reduced proliferation capacity in a concentration-dependent manner (P<0.001) (Fig. 1).

### Cell cycle arrest and apoptosis induced by LBF solution treatment

**Figure 2:**
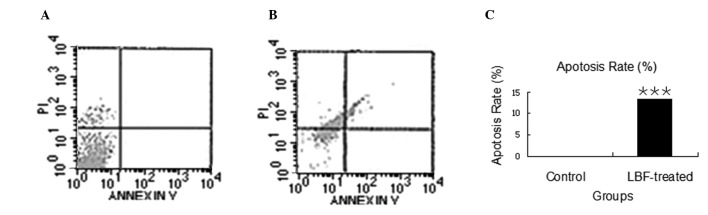
Annexin V/propidium iodide double staining assay. (A and B) Results of flow cytometry analysis of the control and 0.25 mg/ml LBF solution-treated groups; lower left quadrant indicates surviving cells and the lower and upper right quadrants indicate the early and late apoptotic cells, respectively. Upper left quadrant indicates necrotic cells, lower left quadrant indicates surviving cells and the lower and upper right quadrants indicate the early and late apoptotic cells, respectively. Images are representative of the results from two independent experiments. (C) Bar graph demonstrating the results of the flow cytometry analysis evaluating the apoptotic rate of cells. Data are presented as the mean ± SEM, from three independent experiments. One way analysis of variance with Bonferroni’s multiple comparison test was used for statistical analysis. ^***^P<0.001, vs. untreated control. LBF*, Lactobacillus delbrueckii* fermentation.

Next, it was investigated whether the LBF solution inhibits the proliferation of SW620 cells through cell cycle intervention. The results showed that cells treated with LBF solution were arrested in the G1 phase and that the ratio of cells in the G2/M phase was increased compared with that of the control group (Table I). In addition, the Annexin V assay indicated that the number of apoptotic cells had increased to 13.26% (P<0.001) in the LBF-treated cells compared with that in the control group (Fig. 2). These results indicated that the LBF solution treatment induces cell cycle arrest and apoptosis, which may prevent the growth of colon cancer cells.

### LBF solution induces caspase-dependent apoptosis

**Figure 3:**
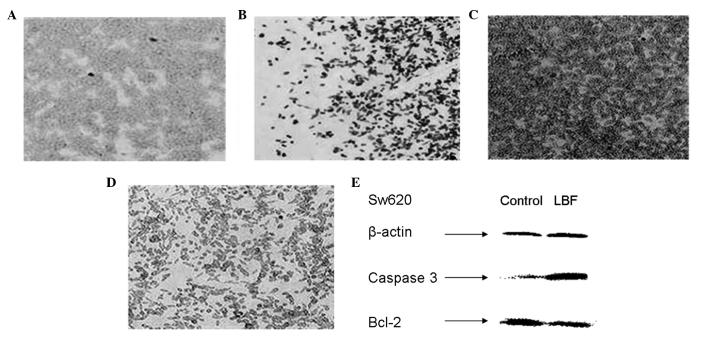
LBF solution induces the activation of the intrinsic apoptotic pathway. Immunohistochemistry images were captured under a microscope (magnification, ×40), of the control and 0.25 mg/ml LBF solution-treated cells stained for (A and B) caspase 3 and (C and D) Bcl-2. (E) Western blot analysis of the SW620 cell lysates treated with and without 0.25 mg/ml LBF solution for caspase 3 and Bcl-2. Uniform loading was confirmed by β-actin and the images are representative of the results from three independent experiments. LBF, *Lactobacillus delbrueckii* fermentation.

The extrinsic and intrinsic apoptotic pathways have been well recognized as major mechanisms of cell death in the majority of cellular systems (8). Therefore, since the results of the current study have shown that the LBF solution induces apoptosis, it was next investigated whether the apoptosis is caspase driven. The expression of caspase 8 was detected in the cells exposed to the LBF solution and the results showed that the LBF solution did not induce caspase 8 activation (data not shown). However, the caspase 3 expression in the colon cells treated with LBF solution was found to increase in the immunohistochemistry assay (Fig. 3A and B). This observation was confirmed by western blot analysis (Fig. 3F) and suggested that the LBF solution induces apoptosis through the caspase 3 intrinsic apoptotic pathway.

Bcl-2 protects cells from apoptosis by preventing the release of mitochondrial cytochrome *c*, therefore, Bcl-2 prevents a caspase 3-dependent proteolytic cascade (9). Furthermore, Bcl-2 expression was detected in the colon cells exposed to the LBF solution. The results indicated that LBF solution decreases Bcl-2 expression, which is likely to contribute to the activation of the caspase 3 intrinsic apoptotic pathway (Fig. 3C, D and F).

### LBF solution inhibits matrix metalloproteinase (MMP)-9 activity

**Figure 4:**
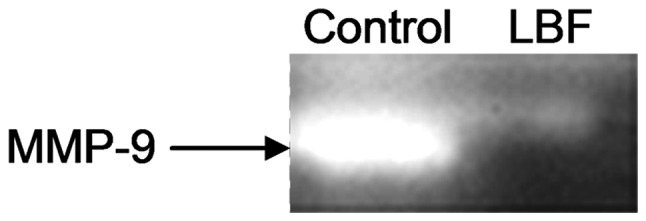
Effects of the LBF solution on the gelatinolytic activity of MMP-9. Gelatin zymography was performed on the untreated and LBF-treated (0.25 mg/ml for 24 h) cells to detect MMP-9 activity. Images are representative of the results from three independent experiments. LBF*, Lactobacillus delbrueckii* fermentation; MMP-9, matrix metalloproteinase-9.

MMPs are overexpressed in various types of cancer and have been associated with tumor invasion due to their capacity to degrade the basement membrane (10). Furthermore, numerous reports have the shown potent invasive activities of several MMPs, including MMP-9 (11). To investigate whether the LBF solution affects the invasive potential of colon cancer cells, MMP activity was evaluated by gelatin zymography assay. The results showed that MMP-9 activity was markedly decreased in cells treated with the LBF solution compared with that of the untreated sample (Fig. 4). This suggested that the LBF solution not only inhibits the proliferation of colon cancer cells, but also antagonizes invasion.

## Discussion

Colon cancer is one of the most common types of cancer in western industrialized countries and environmental factors are significantly involved in its development (12). Certain epidemiological studies have shown a decreased incidence of colon cancer in individuals consuming fermented milk products or yogurt (13). *L. delbrueckii* is usually used for yogurt fermentation; however, whether LBF solution may prevent the growth of colon cancer, and the precise mechanisms underlying this process, are yet to be thoroughly understood. Therefore, the aim of the present study was to investigate the effects of LBF solution on colon cancer SW620 cells *in vitro*.

The results of the present study indicated that the LBF solution causes growth inhibition and induces G1 phase arrest in SW620 cells. Reports have also indicated that the administration of other Lactobacillus strains, such as *L. fermentum* or *L. plantarum* may inhibit colon cancer formation in mouse models (14). In addition, studies have shown that yogurt consisting of milk fermented by the *L. delbrueckii subsp. bulgaricus* strain 2038 and *Streptococcus salivarius subsp. thermophilus* strain 1131 reduced the number and size of colon tumors in RasH2 mice (7).

The present study further investigated the mechanisms of the inhibitory effect of the LBF solution on the growth of colon cancer cells. The observations revealed that the LBF solution inhibits the proliferation of SW620 cells by triggering apoptosis. This insight into the molecular mechanisms of the solution indicated that the LBF solution may significantly increase caspase 3 expression with a markedly reduced expression of Bcl-2, a potent anti-apoptotic protein, in colon cancer SW620 cells. However, the exact compounds in the LBF solution which are responsible for the apoptotic effects on the colon cancer cells remain unclear.

Reports on the role and/or effect of probiotics in the later stages of colon cancer, specifically metastasis, are limited. However, MMP-9, which degrades the basement collagen membrane, has been reported to be a particularly important proteolytic enzyme involved in colon cancer cell invasion (15). The present study revealed that the LBF solution decreases the MMP-9 protein activity in colon cancer cells. In addition, a recent study revealed a similar result in the cell-free supernatant obtained from probiotic *L. caser* and *L. rhamnosus* GG that may decrease MMP-9 activity and inhibit colon cancer invasion (16).

In conclusion, the results of the present study are the first to indicate that the fermentation supernatants of the *L. delbrueckii* strain may efficiently inhibit proliferation and induce apoptosis through the caspase 3-dependent pathway in colon cancer cells. In addition, the fermentation supernatants of the *L. delbrueckii* strain were found to decrease MMP-9 activity in SW620 cells, which may prevent the invasion of colon cancer cells. Unlike chemotherapy, the intake of probiotics are less likely to cause side effects. Therefore, we hypothesize that the use of *L. delbrueckii,* as described in the present study, for the treatment of colon cancer may be beneficial to patients.

## Figures and Tables

**Table I:** Effect of LBF solution on the cell cycle of SW620 cells.

	Proportion of cells in each phase, %			
	
Treatment	Sub-G1	G1	S	G2-M
Control	1.97	43.90	52.48	1.65
LBF	2.04	64.72	27.85	5.39

Following treatment with LBF solution (0.25 mg/ml) for 24 h, the cells were harvested, stained with propidium iodide and the proportion of cells in each phase of the cell cycle was measured. LBF, *Lactobacillus delbrueckii* fermentation.

## Abbreviations

LBF: *Lactobacillus delbrueckii* fermentation
FBS: fetal bovine serum
PI: propidium iodide
PBS: phosphate-buffered saline
MTT: 3-(4,5-dimethylthiazol-2-yl)-2, 5-diphenyltetrazolium bromide
MMPs: matrix metalloproteinases
